# Remarkable disjunctions in *Ipomoea* species (Convolvulaceae) from NE Brazil and Central America and their taxonomic implications

**DOI:** 10.1007/s12225-017-9710-9

**Published:** 2017-09-21

**Authors:** John R. I. Wood, Maria Teresa Buril, R. W. Scotland

**Affiliations:** 1 Department of Plant Sciences, University of Oxford, South Parks Road, Oxford, OX1 3RB UK; 2 Royal Botanic Gardens, Kew, Richmond, Surrey TW9 3AB UK; 3 Department of Biology, Universidade Federal Rural de Pernambuco, Rua Dom Manoel de Medeiros s.n., Recife, PE 52171-030 Brazil

**Keywords:** Altos de Campana, Bolivia, Brejo de Altitude, Chagres National Park, Costa Rica, disjunct distribution, inselbergs, Mata Atlântica, Panama, taxonomy

## Abstract

Recent collections of *Ipomoea* from North East Brazil have revealed a number of unexpected disjunct distributions. The most remarkable is that of *I. eremnobrocha* D. F. Austin, previously thought to be endemic to Panama but now known from three states in NE Brazil. Revision of Panamanian material named *I. eremnobrocha* unexpectedly showed that two distinct species had been treated under this one name. Specimens from the Chagres National Park area in Panama are described as a new species under the name *I. isthmica* J. R. I. Wood & Buril while *I. eremnobrocha* is retained as the correct name for the plant from the Altos de Campana in Panama and NE Brazil. An amended description of this species is given and a table of differences between the related species is provided. Two recently described species from Bolivia, *I. graniticola* J. R. I. Wood & Scotland and *I. chiquitensis* J. R. I. Wood & Scotland are recorded from NE Brazil several thousand km from their type localities. Attention is drawn to the role of granite inselbergs as sites of species with a disjunct distribution. A possible relative of *I. chiquitensis* is described as a new species from NE Brazil under the name *I. melancholica* J. R. I. Wood & Buril. The new species are illustrated with line drawings and maps of the unusual distribution patterns are provided.

## Introduction

Northeastern Brazil is drier than other parts of the country and much of it is covered in various kinds of dry forest and, in particular, with deciduous, often spiny woodland known as caatinga. There are exceptions to this picture. South from approximately 7°S and running parallel to the coast are relics of Mata Atlântica or Brazilian Atlantic Forest (Nogueira Rodal *et al.* 2005), which in some areas penetrates into the more widespread caatinga of the region. The Mata Atlantica itself is not uniform but consists of a mosaic of different physiognomies including deciduous woodland and, at higher elevations with greater rainfall, a distinctive vegetation known as “Brejo de Altitude”, sometimes translated as “northeastern upland forest” (Porto *et al.* 2004). As in other parts of Brazil diverse species of *Ipomoea* L. are present and it was from here that the authors found two putative new species which are discussed in this paper and whose distribution and identity has extensive ramifications.

## Materials and Methods

Our results are based on the examination of dried material principally from herbaria in Brazil and the United States. We have been able to compare specimens with a wide range of material from elsewhere as part of on-going monographic work on *Ipomoea* being carried out at the University of Oxford. We have also made use of on-line images, principally from Jstor http://plants.jstor.org. and from the herbarium of the Universidad de Panama http://herbario.up.ac.pa/Herbario/herb/vasculares/view.species/1392. The Tropicos data base http://www.tropicos.org/ has also provided useful information. Field work in Brazil was carried out by Teresa Buril and students. Molecular sequencing of samples was carried out at the Department of Plant Sciences at Oxford.

## The identity of *Ipomoea eremnobrocha*

One of the putative new species was quite unlike any other species recorded from Brazil. It had deeply 3-lobed leaves which were sericeous on the lower surface, a shortly pedunculate, compact cymose inflorescence with a cream-coloured campanulate corolla and densely woolly seeds. This suggested an affinity with the Mesoamerican species *Ipomoea peteri* (Kuntze) Staples & Govaerts but this could be easily distinguished by its larger, pink, funnel-shaped corolla and by the much longer outer and inner sepals. We were confident that we had a very distinct new species.

We had not carefully compared the putative new species with *Ipomoea eremnobrocha* D. F. Austin, described from Panama, because the entire-leaved plant illustrated with the protologue (Austin 1997: 152) was so obviously different from the plant in NE Brazil. It was only when the first author obtained a loan from Missouri (MO) as part of Oxford University’s monographic studies of *Ipomoea*, that it was realised that specimens from Panama identical to the Brazilian plant had been named *I. eremnobrocha* by Austin. A further loan made it clear that Austin had labelled specimens of two separate species from Panama with this name and that the protologue of *I. eremnobrocha* was based on elements of both species.

It was accordingly necessary to see the type of *Ipomoea eremnobrocha* (*A. Gentry* 5759) in order to decide which species the name was linked with. Here, however, another complication arose as the holotype could not be found at MO, nor the isotype at ARIZ or FTG, to which Austin’s Convolvulaceae specimens had been transferred from FAU. No duplicate was present at PMA either. It must be assumed that the original material was never returned to Missouri and has somehow been mislaid during the successive transfers of Austin’s Convolvulaceae from FAU to FTG and thence to ARIZ.

**Map 1:**
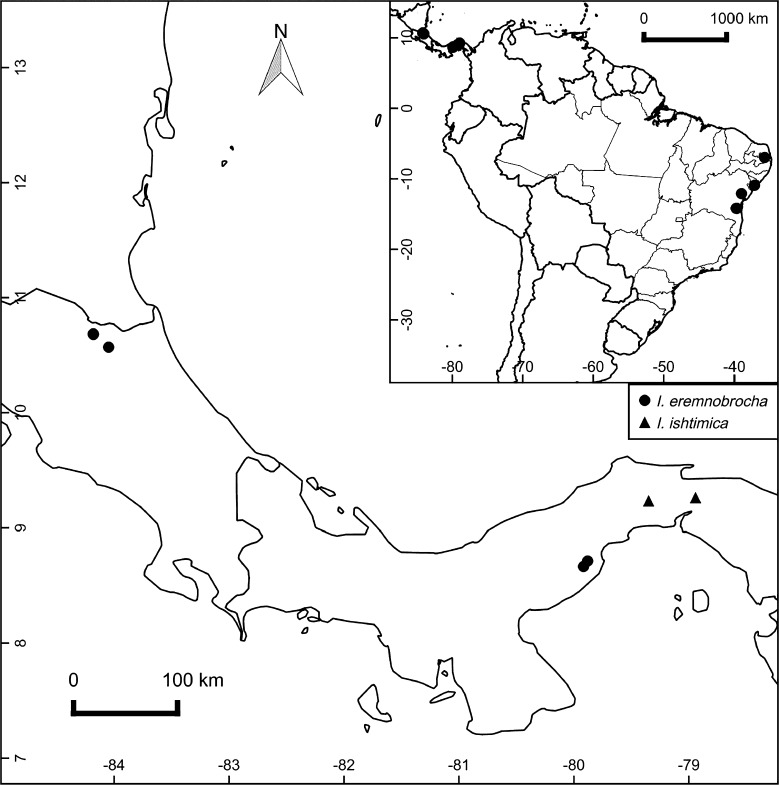
Distribution of *Ipomoea isthmica* (▲) and *I. eremnobrocha* (●) in Brazil and Central America with inset of distribution in Panama.

Despite the apparent loss of the type we can be certain that it corresponds to the species with a 3-lobed leaf as all specimens coming from the type locality, Cerro Campana, are of this plant. Specimens of the entire leaved plant, in contrast, were all collected from east of Panama City on or around Cerro Jefe in the Chagres National Park (Map 1). As the Chagres plant is clearly a different species we are describing it as new:

**Ipomoea isthmica** *J. R. I.Wood & Buril,* **sp. nov.** Type: Panama, Prov. Panama, Cerro Jefe, 22 Sept. 1972, *Al. Gentry* 6135 (holotype MO).

http://www.ipni.org/urn:lsid:ipni.org:names:77165111-1

**Fig. 1:**
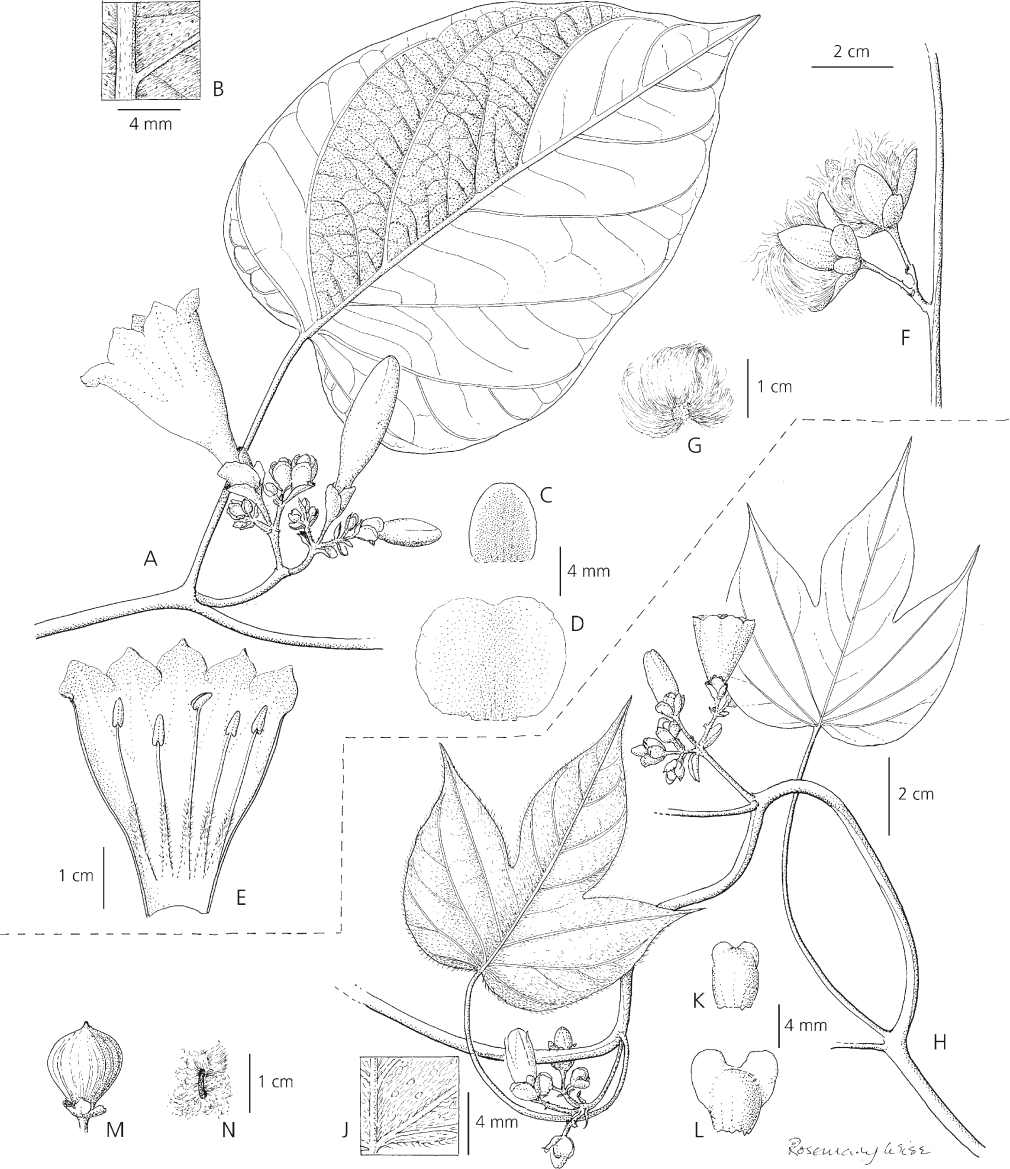
** A – G** *Ipomoea isthmica*. **A** habit showing leaf and inflorescence; **B** abaxial leaf surface; **C** outer sepal; **D** inner sepal; **E** corolla opened up to show stamens; **F** fruiting cyme; **G** seed. **H – N** *Ipomoea eremnobrocha*. **H** habit showing leaves and inflorescence; **J** abaxial leaf surface; **K** outer sepal; **L** inner sepal; **M** capsule; **N** seed. **A – E** from *Nee* 7912; **F – G** from *McPherson & Merello* 8202; **H – J** from *Polo* 39; **K – L** from *D’Arcy* 9551; **M – N** from *Correa et al*. 11312. drawn by rosemary wise.

Perennial *liana* reaching 8 m in height, stems woody, thinly pubescent, purplish-brown. *Leaves* petiolate, 7 – 15 × 6 – 11 cm, ovate, ovate-deltoid or suborbicular, truncate to very broadly cuneate with rounded auricles, apex very shortly acuminate and mucronulate, acute or retuse, margin entire to obscurely denticulate, adaxially green, glabrous or sparsely and softly strigose, abaxially densely silvery-sericeous, punctate, the venation prominent; petioles 3.5 – 8 cm, terete, pubescent. *Inflorescence* of compact axillary cymes with 3 – 10 flowers; primary peduncles 0.8 – 2 cm, grey-sericeous; bracteoles 2 – 3 × 0.5 mm, linear, obtuse, somewhat scarious, puberulent, tardily deciduous; secondary peduncles 3 – 5 mm, puberulent; pedicels 5 – 15 mm, puberulent below, becoming glabrous and thickened upwards; sepals unequal, glabrous, outer 5 – 7 × 4 – 6 mm, ovate to obovate, rounded, margins narrow, scarious, inner sepals 8 – 11 mm long and wide, suborbicular, rounded to retuse, the margins broad, scarious; corolla funnel-shaped, pale magenta towards the apex but dark purple basally, glabrous on the exterior, 4.5 – 5.5 cm long; limb c. 3.5 cm diam., apparently weakly lobed; stamens included; filaments inserted just above the corolla base, glabrous except for the densely pubescent base, longer pair c. 22 mm, shorter c. 15 mm, anthers 5 mm long; style c. 25 mm, glabrous, stigma biglobose; ovary broadly conical, 1 – 2 mm long, glabrous. *Capsule* 18 – 20 × 12 – 15 mm, ovoid, very shortly apiculate with persistent style base, glabrous, 4-seeded; *seeds* c. 5 × 2.5 mm, dark brown, broadly oblong in outline, densely lanate with matted brownish cottony hairs up to 15 mm long. Fig. 1A – G.

**recognition**. *Ipomoea isthmica* is a distinctive species not readily confused with any other species from Mesoamerica because of its liana habit, large, abaxially sericeous, entire leaves and shortly pedunculate compact inflorescence with a funnel-shaped magenta corolla and woolly seeds. It might be compared with *I. steerei* (Standl.) L. O. Williams as both species have entire leaves but the relatively small (<6 × 3.5 cm) oblong-elliptic, strongly apiculate leaves of *I. steerei* are very different from the ovate to suborbicular, acute, retuse to shortly acuminate leaves of *I. isthmica* which are 7 – 15 × 6 – 11 cm in size. Additionally, the lax few-flowered inflorescence of *I. steerei* is quite different. *I. isthmica* is distinguished from *I. eremnobrocha* by the much larger, entire, not 3-lobed leaves, the longer very unequal sepals and the longer pale magenta corolla with a dark purple base. These differences can be observed in the comparison plate, Fig. 1, and in Table 1. Vegetatively *I. isthmica* appears most similar to *I. cuprinacoma* E. Carranza & J. A. McDonald in the size and shape of the leaves and in the distinct shape of the sepals. However *I. isthmica* differs in the compact, up to 10-flowered cymes with short peduncles and pedicels, relatively persistent bracteoles, the suborbicular inner sepals and the magenta corolla with blackish throat. In contrast *I. cuprinacoma* has relatively lax 1 – 3 (– 5)-flowered cymes with pedicels up to 25 mm long, early caducous bracteoles, obovate inner sepals and a slightly larger (to 8 cm long) white corolla with bluish throat.

**Table 1.** Differences between *Ipomoea isthmica*, *I. eremnobrocha*, *I. peteri* and *I. steerei.*

	*I. isthmica*	*I. eremnobrocha*	*I. peteri*	*I. steerei*
Flowering stem	Relatively stout, c. 3 mm thick	1.5 – 2 mm thick	2 – 2.5 mm thick	1.5 – 2 mm thick, conspicuously twining
Leaf shape	Entire, ovate to suborbicular	3-lobed, broadly ovate	3-lobed (? rarely entire), broadly ovate	Entire, oblong to elliptic
Leaf size (cm)	7 – 15 × 6 – 11	5 – 12 × 7 – 12	4 – 14 × 3 – 12	3 – 6 × 2 – 3.5
Leaf apex	Retuse, acute or short-acuminate, mucronulate	Long-acuminate, mucronate	Obtuse, strongly mucronate	Obtuse, strongly apiculate
Leaf base	Truncate or subcordate, briefly cuneate	Cordate, briefly cuneate	Cordate, sometimes briefly cuneate	cuneate
Adaxial surface	Nearly glabrous	Appressed pubescent	Appressed pubescent	Appressed pubescent
Abaxial surface	Sericeous, veined prominently, glands darkish	Sericeous, glands white	Sericeous, glands white	Sericeous, glands white
Appearance of inflorescence	Dense, many-flowered	Dense, many-flowered	Dense, many-flowered	Lax, usually 1 – 2-flowered
Peduncle relative to pedicel	Stout	Stout	Stout	Similar but densely sericeous
Outer sepals	Glabrous, 5 – 7 mm long, rounded	Glabrous except at base, 4 – 5 mm long, truncate	Shortly pilose, 7 – 10 mm long, acute	Glabrous, 6 – 7 mm long, rounded
Inner sepals	8 – 11 mm long, suborbicular, rounded	5 – 6 × 4 – 5 mm, obovate, strongly retuse	8 – 11 × 4 – 5 mm, oblanceolate to obovate, rounded to retuse	8 – 10 (– 12) × 6 mm obovate, rounded to retuse
Corolla shape	4.5 – 5.5 cm, funnel-shaped	2 – 2.5 cm, subcampanulate	4.5 – 5 cm, funnel-shaped	5 – 5.5 cm, funnel-shaped
Corolla colour	Magenta with greenish tube and blackish throat	Creamy-white	Pinkish-purple	Pinkish-purple
Capsule	Ovoid, 18 – 20 mm long	Subglobose to ellipsoid, 12 – 13 mm long	Subglobose, 8 – 10 mm long	Ovoid, 12 – 15 mm long

**illustrations**. There is a good illustration of *Ipomoea isthmica* accompanying the original protologue of *I. eremnobrocha* (Austin 1997: 152). All the elements in this plate and the specimens from which they were drawn are clearly attributable to *I. isthmica*. Both specimens imaged in Jstor http://plants.jstor.org. (accessed on 4 Oct. 2016) under *I. eremnobrocha* are *I. isthmica*.

**distribution**. Endemic to Panama. Restricted to the area of the Chagres National Park east of Colon and Panama City. Most collections are from the Cerro Jefe area.

**specimens examined** **.** **panama**. Prov. Panama. Cerro Jefe, 2700 ft, 9 July 1966 fl., *E. L. Tyson et al.* 4292 (MO); ibid., c. 1000 m, 22 Sept 1972 fl., fr., *A. Gentry* 6135 (holotype MO); ibid., 850 – 900 m, 29 Oct. 1980 fl., *K. J. Sytsma* 2018 (MO); El Llano-Carti road 5 km N of Pan American highway at El Llano, 300 m, 10 Nov. 1973 fl., *M. Nee* 7912 (MO); ibid., 7 km from Pan American highway, 9°15'N 79°00'W, 400 m, 28 Jan. 1986 fl., fr., *G. McPherson & M. Morello* 8202 (MO).

There are many other records in Tropicos http://www.tropicos.org/ (accessed on 4 October 2016) from the Cerro Jefe area which almost certainly belong to this species including *G. A. Sullivan* 203 (MO), *M. D. Correa et al.* 1587 (MO), *R. L. Dressler* 3040 (MO), *J. P. Folsom & R. L. Hartman* 4635 (MO), *K. J. Sytsma* 1466 (MO); *W. H. Lewis & R. L. Dressler* 7554 (MO); *J. D. Dwyer* 8506 (MO). The description, in most cases, of the corolla as pink with a dark centre, rather than white confirms this. Folsom’s description on the collection label as “Corolla antique pink turning green to a black throat” is particularly descriptive. The record from nearby Cerro Azul (*D. M. Porter et al*. 4075 (MO)) and records from the Santa Rita Ridge (*M. D. Correa & R. L. Dressler* 976 (MO); *J. D. Dwyer & A. Gentry* 9403 (MO)) are also probably this species.

**habitat**. The habitat of *Ipomoea isthmica* is variously described as “premontane wet forest” or “cloud forest” and it was collected on forest edges and in roadside scrub between 300 and 1000 m.

**conservation status**. Although geographically restricted, all or most of the c. 20 records of this species are from the Chagres National Park, which enjoys legal protection. The new species favours disturbed habitats so should face no significant threat. However in the complete absence of any population studies, this species can only be categorised as Data Deficient (DD) until such studies can be carried out.

**notes**. We have not yet been able to sequence material of this species but its morphology suggests it belongs to the small clade with *Ipomoea steerei*, which is discussed below.

As the protologue of *I. eremnobrocha* (Austin 1997: 145ff.) combines elements of *I. isthmica* with *I. eremnobrocha*, we are redescribing the former with an amended description as follows:

**Ipomoea eremnobrocha** *D. F. Austin* (1997: 145), emend. *J. R. I. Wood & Buril*. Type: Panama, Cerro Campana, *A. Gentry* 5759 (holotype cited from MO and isotype cited from FAU, but both missing).

Perennial *climber* or *liana* of unknown height but reaching at least several metres high, stems twining, somewhat woody below, herbaceous above, thinly pubescent when young, glabrescent, pale brown. *Leaves* petiolate, ovate-truncate in outline, 5 – 12 × 7 – 12 cm, 3-lobed to about half way, base ± truncate or subcordate and shortly cuneate onto the petiole, apex finely acuminate and shortly mucronate, central lobe oblong-elliptic (rarely ovate), 2 – 5 × 2 – 4 cm, laterals broadly ovate, margins entire or undulate, adaxially green, pubescent, abaxially densely silvery-sericeous with appressed hairs and scattered white glands; petioles terete, 4 – 11 cm, thinly pubescent. *Inflorescence* of compact axillary cymes with up to about 10 flowers; primary peduncles 0.5 – 2.5 cm, stout, pubescent; bracteoles 2 – 7 × 0.5 – 1 mm, filiform to lanceolate, acuminate, pubescent, tardily deciduous; secondary peduncles 3 – 5 mm; pedicels 3 – 8 mm, pubescent; sepals somewhat unequal, outer 4 – 5 × 2 – 3 mm, broadly oblong, truncate or slightly retuse, glabrous or with a few hairs at the base, inner 5 – 6 × 3 – 4 mm, obovate, usually strongly retuse with a broad sinus so appearing winged, margins scarious; corolla ±campanulate, white, glabrous on the exterior, 2 – 2.5 cm long; limb 2.2 – 2. 5 cm diam., stamens included; filaments inserted just above the corolla base, glabrous except for pubescent base, longer 9 mm, shorter 8 mm, anthers 2 mm long; style 15 – 18 mm, glabrous, stigma biglobose; ovary glabrous. *Capsule* 12 – 13 × 10 – 11 mm, ellipsoid to subglobose, very shortly apiculate with persistent style base, glabrous, 4-seeded; *seeds* 6 × 1.5 – 2 mm, brown, broadly oblong in outline, densely lanate with matted cottony hairs up to 10 mm long. Fig. 1H – N.

**recognition**. *Ipomoea eremnobrocha* is most closely related to *I. peteri*, which is widely known under the name *I. tuxtlensis* House. Austin (1997) correctly compared it with this species as both have 3-lobed leaves, abaxially white and dotted with white glands combined with compact, shortly pedunculate inflorescences with somewhat persistent lanceolate-filiform bracteoles and woolly seeds. *I. peteri* can be distinguished easily by its larger (c. 5 cm long), funnel-shaped pink corolla and the subequal, densely hirsute sepals. Austin also compared *I. eremnobrocha* with *I. steerei* but this species is immediately distinguished by its oblong-elliptic, entire leaves, lax, few-flowered inflorescence and relatively large, funnel-shaped pink corollas c. 5 cm in length. *I. isthmica* described above is also readily distinguished by the large, ovate to suborbicular, entire leaves, longer sepals and the larger funnel-shaped pale magenta corolla with a dark tube. The 3-lobed leaves with finely attenuate-acuminate tips, short, basally pubescent sepals and the smaller subcampanulate, cream-coloured corolla of *I. eremnobrocha* are very distinct. Table 1.

**illustrations**. There are excellent photographs of this species by Mireya D. Correa and Fermín Hernández on the website http://herbario.up.ac.pa/Herbario/herb/vasculares/view.species/1392 (accessed 21 March 2017). All specimen images of *Ipomoea eremnobrocha* at http://fm1.fieldmuseum.org/vrrc (accessed 21 March 2017) are also of this species.

**distribution**. *Ipomoea eremnobrocha* has a very disjunct distribution being found in NE Brazil, Panama and neighbouring Costa Rica. In Brazil it is present in three north eastern states: Bahia, Paraiba and Sergibe (Map 1). The records from Costa Rica are included provisionally as they are of sterile material although this is apparently correctly identified. 

**specimens examined** **.** **brazil**. **Bahia**: Litoral Sul, Itagibá, Campo Verde, 14°10'02"S 39°43'20"W, 15 Sept. 2008, *C. E. Ramos, L. J. Alves & C. E. Santos* 397 (ALCB); ibid., Mata da Botinha, 14°10'53"S 39°42'31"W, 2009, *M. L. Guedes et al.* 16520 (ALCB, US); Muritiba, estrada para São Felipe 4 km, 12°05'S 39°02'W, 22 May 2003, *E. C. Schmidt et al.* 313 (HUEFS). **Paraiba**: Mun. Areia, Mata do Pau Ferro, 28 May 1980, *Andrade-Lima, Fevereiro & Pereira* 01 (IPA, OXF). **Sergipe**: São Cristóvão, Mata da Escola Agrotécnica Federal, 15 Aug. 1997, *M. Landim et al.* 1316 (ASE7882). **costa rica**. Provisional records based on sterile specimens: Alajuela: Cantón de San Carlos, c. 7 km NE de Boca Tapada, Lagarto Lodge, 10°41'10"N 84°10'50"W, 90 m, 27 July 1996, *B. Hammel* 20340 (CR, INB, MO). Heredia: Cantón de Sarapiquí, Lomas Sardinal, c. 15 km linea recta N de Puerto Viejo, 10°34'10"N 84°02'50"W, 250 – 350 m, 19 Jan. 1997, *B. Hammel* 20688 (CR, INB, MO). **panama**. Prov. de Panama: Cerro Campana, 12 Nov. 1975 fl., *W. G. D’Arcy* 9551 (MO); ibid., fr., *W. G. D’Arcy* 9592 (MO); Campana, hacia la carretera de Chica, 1150 m, 23 Nov. 1975 fl., *C. E. Polo* 39 (MO, PMA); Altos de Campana, 110 metros del Motel Sulin, 3060 ft, 12 Nov. 1977 fr., *R. Méndez* 57 (MO, PMA); Cerro Campana, savannas south of radio tower, 10 Nov. 1978, fr., *B. Hammel* 5519 (MO); P.N. Altos de Campana, sendero de Interpretación, 1 km al este del campamento de los guardebosques, 8°40'N 79°55'W, 800 – 900 m, 25 Aug. 1994, fl., *M. D. Correa & E. Montenegro* 10760 (PMA); cerca de las antiguas instalaciones del Motel Sulim, 8°40'N 79°55'W, 800 – 900 m, 26 Oct. 1995 fr., *M. D. Correa et al.* 11312 (F, MO, PMA). Additionally the following specimens cited at http://herbario.up.ac.pa/Herbario/herb/vasculares/view.species/1392 (Accessed 4 Oct. 2016) are almost certainly this species: *R. Aizprúa* B1909 (PMA), 2179 (PMA); *FLORPAN* 2282 (PMA); *F. Hernández* 693; *C. Galdames* 1817 (PMA); *M. D. Correa et al.* 8074 (PMA), 9300 (PMA), and 10903 (PMA).

**habitat**. In Panama “Bosque húmedo tropical premontano” (*Correa et al.* 11312), roadsides, trailsides and similar disturbed bushy areas disturbed places between 350 – 1100 m. In Brazil it is recorded as growing in deciduous forest, “mata estacional decidual” (*Guedes et al.* 16520) or woodland on damp clay soil (*Andrade-Lima et al.* 01). This last is from an area of Brejo de Altitude vegetation. No altitude details are known. If correctly identified the Costa Rican plants grow on forest margins at very low altitudes of c. 100 – 350 m.

**note**. It is unfortunate that the epithet “*eremnobrocha*” has to be used for this species as it is based on the Greek “eremnos” = black and “brochtos” = throat (Austin 1997: 148) which is very appropriate for *Ipomoea isthmica* but quite inappropriate for this species.

At Oxford we have been able to sequence material of *Ipomoea eremnobrocha* using *ITS* from *M. D. Correa et al.* 11312 and this shows that it belongs to a well-supported clade with *I. peteri*, *I. steerei* and *I. heterodoxa* Standl. & Steyerm., all four species from Mesoamerica. Given its morphology, it is likely that *I. isthmica* also belongs to this clade. The differences between these four species are shown in Table 1.

## A note on disjunct distributions

The most striking disjunctions in neotropical *Ipomoea* are those of a number of relatively common species with a distinct amphitropical distribution, something only occasionally commented on in the literature, for example McDonald 1995: 112. These species are centred in the northern hemisphere on Mexico and the United States and in the southern hemisphere on Bolivia, Argentina and Paraguay. They are absent from all or most of the intervening territory. Among species showing this kind of distribution are *I. amnicola* Morong, *I. cordatotriloba* Dennst., *I. crinicalyx* S. Moore, *I. plummerae* A. Gray and *I. pubescens* Lam. Although there is some suspicion that the first two were introduced over part of their range, there is no dispute over the status of the other three.

A unique disjunct distribution is that of *Ipomoea subrevoluta* Choisy. It is entirely restricted to the South American continent apart from single occurrences on the island of Trinidad and Isla de Juventud (or Pinos, as it was formerly known) in Cuba. As it is a species of semi-aquatic habitats it might be an ancient introduction to Cuba by birds. No such explanation seems possible for the disjunct distribution of *I. eremnobrocha*. The limited molecular data suggest this species is of Mesoamerican origin and its presence in NE Brazil cannot be explained at the present time by any of the scenarios suggested, for example, by Gentry (1982). We are unaware of any similar distribution pattern except that of *Jacquemontia gracillima* (Choisy) Hallier f. which occurs in scattered populations in NE Brazil (and also in eastern Bolivia) but is only known in Central America from a single collection (*H. Pittier* 4842 (F)) made close to but not in the Altos de Campana National Park in Panama.

Two other species of *Ipomoea* have been discovered in NE Brazil recently, both described from Bolivia in 2015 (Wood *et al.* 2015). *I. graniticola* known from two granite domes in Bolivia and a quartzite outcrop in Mato Grosso (Wood *et al.* 2015) has recently been found in the State of Ceará (*E. B. Souza et al.* 3395 (HUVA, PEUFR,) in a very similar habitat. *I. chiquitensis*, which was known from a single granite rock platform near San Rafael in Eastern Bolivia, has also been found in Ceará (*J. A. A. M. Lourenço* 124 (PEUFR)). Although, the disjunction in these two cases is of almost 3000 km, it is not quite so surprising. The rock domes and platforms which are the habitat of these species function as inselbergs and harbour assemblies of plants very different from the surrounding vegetation but similar in composition to other inselbergs several hundred km away. The remarkable feature in this case is the distance between the known populations. In passing it is worth noting that another species of *Ipomoea*, *I. caloneura* Meisn., occurs on isolated inselbergs in Eastern Bolivia and neighbouring parts of Mato Grosso. It has not yet been found in NE Brazil.

The other new species from NE Brazil shows some morphological similarities with *Ipomoea chiquitensis* although we cannot confirm the link as we have been unable to sequence material of this species which is described below:

**Ipomoea melancholica** *J. R. I. Wood & Buril*, **sp. nov.** Type: Brazil, Alagoas, Quebrangulo, REBIO Pedra Talhada, 6 Sept. 2012, *B. S. Amorim, J. L. Costa-Lima, W. M. Pora, V. S. Sampaio, M. A. Chagas* 1658 (holotype JPB, isotype UFP).

http://www.ipni.org/urn:lsid:ipni.org:names:77165123-1

**Fig. 2:**
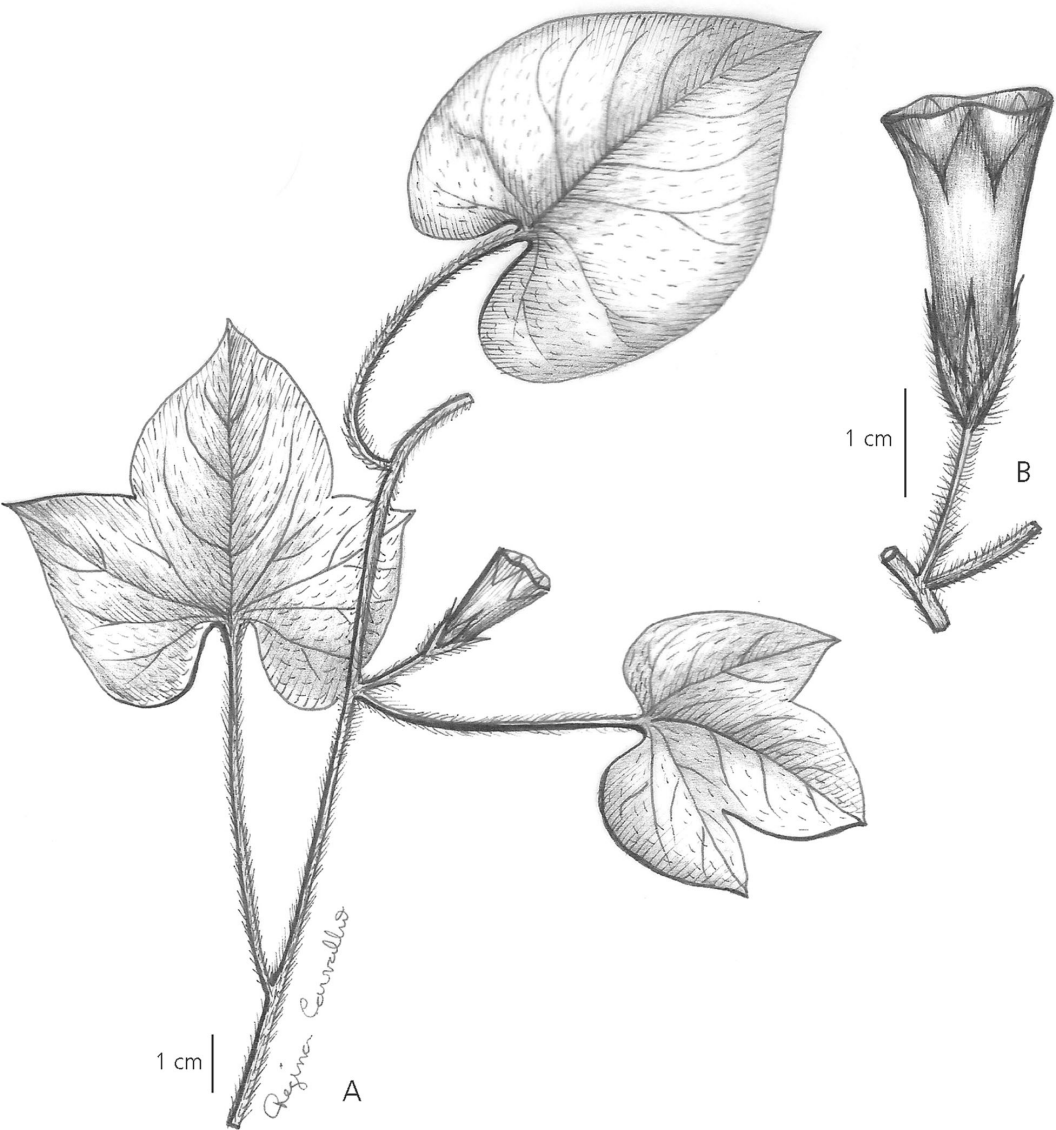
** A – B** *Ipomoea melancholica* **A** habit; **B** inflorescence showing sepals and corolla. **A – B** from *B. S. Amorim et al*. 1658. drawn by regina carvalho.

Slender twining *herb* of unknown height; stems pilose. *Leaves*, ovate and entire 4.5 – 10 × 3.5 – 8.5 cm, undulate to shallowly 3-lobed, base cordate with rounded auricles, apex shortly acuminate, obtuse and mucronulate, adaxially thinly pubescent, abaxially paler, glabrous; margins ciliolate; petioles 1 – 8.5 cm pilose. *Inflorescence* of solitary, pedunculate flowers from the leaf axils; peduncles 3 – 11 mm; bracteoles 1 – 2 mm, lanceolate; pedicels 9 – 13 mm, thickened upwards, slightly winged, often recurving, thinly pubescent; sepals subequal, 7 – 8 × 1.75 mm, lanceolate, acuminate, pubescent and ciliate; corolla 2.5 – 3 cm, pink, narrowly funnel-shaped, apparently glabrous, midpetaline bands ending in small teeth, limb c. 1.5 cm diam.; style globose. *Capsule* 10 × 7 mm, ovoid, shortly rostrate, glabrous; seeds 4, 5 × 2.5 mm, grey, densely tomentose. Fig. 2A, B.

**recognition**. This species has been interpreted as a form of *Ipomoea acanthocarpa* (Choisy) Aschers & Schweinf. (=*I. piurensis* O’Donell) but differs in the solitary flowers and very shortly rostrate capsule. It has also been identified as *I. minutiflora* (M. Martens & Galeotti) House but differs in its larger solitary pink flowers and larger capsule, 10 mm, not 3 mm in length. It might also be thought to be a depauperate species from the *Pharbitis* group such as *I. indica* (Burm.) Merr. but the 4-seeded capsule and small sepals rule that out. The species seems closest to *I. chiquitensis*, a similarly modest, usually solitary-flowered species with a small corolla. Both species have leaves adaxially pubescent but abaxially glabrous, and both have similar-sized, acuminate sepals with white margins as well as deflexed fruiting peduncles. However, *I. chiquitensis* always has entire leaves, the stem is glabrous but the leaves and sepals are much more hirsute, and the capsule is much more prominently rostrate.

**Map 2:**
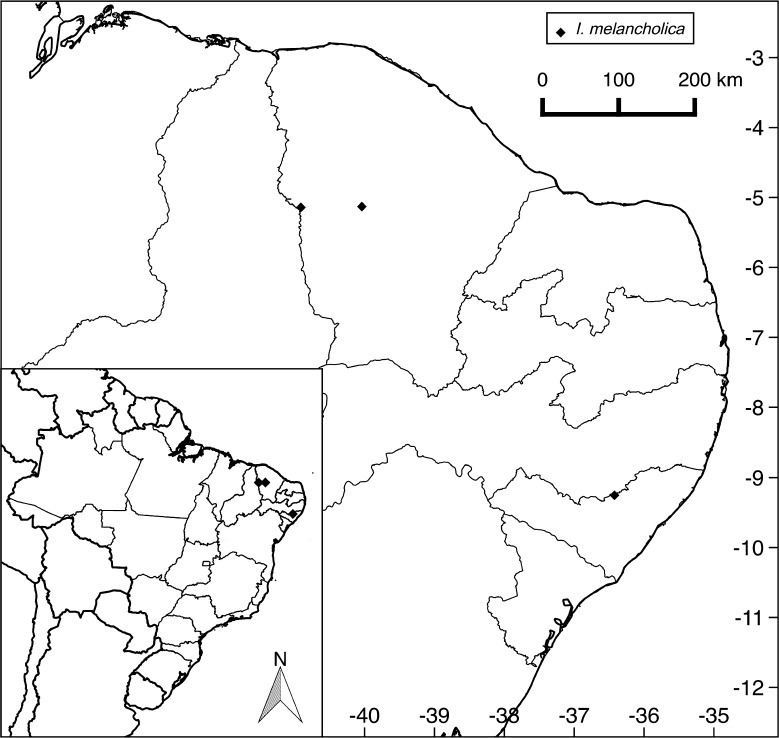
Distribution of *Ipomoea melancholica* (♦) in NE Brazil.

**habitat** **&** **distribution**. Recorded from two states and three localities in northeastern Brazil (Map 2). very little is known about its habitat but the type appears to have been collected around a rock outcrop in Atlantic Forest, a similar habitat to that of *Ipomoea chiquitensis*. It grows in dry forest fide *Araujo* 1424.

**specimens examined**. **brazil**. **Alagoas**: Quebrangulo, REBIO Pedra Talhada, 9°15'16"S 36°25'50"W, 6 Sept. 2012, *B. S. Amorim et al.* 1658 (JPB, UFP); ibid., *G. A. Gomes-Costa* 166 (JPB, UFP). **Ceará**: [possibly Independencia, Serra de Baturité] Sitio B, Inácio de Azevedo. 17 June 1937, *J. Eugenio* 1007 (GH); Mun. Crateús, Serra das Almas, 5°08'31"S 40°54'45"W, 600 m, 7 May 2002, *F. S. Araujo* 1424 (EAC, HUEFS).

**conservation status**. With only three relatively recent collections this species appears to be rare despite its presence in two states. However, it can only be classified as Data Deficient (DD) within IUCN (2012) guidelines until its populations can be studied in detail.

**etymology**. The epithet *melancholica* (“sad”) refers to the drab appearance of this species with its solitary flowers, usually bent-over in fruit, thus presenting a rather forlorn aspect.

## References

[CR1] Austin DF (1997). Dissolution of *Ipomoea* series *Anisomerae* (Convolvulaceae). J. Torrey Bot. Soc..

[CR2] Gentry, A. H. (1982). Neotropical Floristic Diversity: Phytogeographical connections between Central and South America, pleistocene climatic fluctuations, or an accident of Andean orogeny. *Ann. Missouri Bot. Gard.* 69: 557 – 593.

[CR3] IUCN (2012). *Guidelines for application of IUCN Red List Criteria at Regional and National Levels*. International Union for the Conservation of Nature, Gland.

[CR4] McDonald JA (1995). Revision of *Ipomoea* section *Leptocallis* (Convolvulaceae). Harvard Pap. Bot..

[CR5] Nogueira Rodal MJ, Ferreira Sales M, da Silva MJ, da Silva AG (2005). Flora de um Brejo de Altotude na escarpa oriental do planalto da Borborema, PE, Brasil. Acta Bot. Bras..

[CR6] Porto, K. C., Cabral, J. J. P. & Tabarelli, M. (eds) (2004). *Brejos de Altitude em Pernambuco e Paraíba*. Ministério do Meio Ambiente, Brasilia, D.F.

[CR7] Wood JRI, Carine MA, Harris D, Wilkin P, Williams B, Scotland RW (2015). *Ipomoea* (Convolvulaceae) in Bolivia. Kew Bull..

